# Patient-centered, comparative effectiveness of esophageal cancer screening: protocol for a comparative effectiveness research study to inform guidelines for evidence-based approach to screening and surveillance endoscopy

**DOI:** 10.1186/1472-6963-12-288

**Published:** 2012-08-28

**Authors:** Jennifer R Kramer, Jennifer Arney, John Chen, Peter Richardson, Zhigang Duan, Richard L Street, Marilyn Hinojosa-Lindsey, Aanand D Naik, Hashem B El-Serag

**Affiliations:** 1 Houston VA HSR&D Center of Excellence, Michael E. DeBakey Veterans Affairs Medical Center, Houston, TX, USA; 2 Department of Medicine, Baylor College of Medicine, Houston, Texas, USA; 3 Department of Communications, Texas A&M University, College Station, TX, USA; 4 Department of Sociology, University of Houston-Clear Lake

**Keywords:** Barrett’s esophagus, Endoscopy, Mixed methods research, Comparative effectiveness research

## Abstract

**Background:**

The comparative effectiveness (CE) of endoscopic screening (versus no screening) for Barrett’s esophagus (BE) in patients with GERD symptoms, or among different endoscopic surveillance strategies in patients with BE, for the early detection of esophageal adenocarcinoma (EA) is unknown. Furthermore, it is unclear if patients or providers have or will adopt any of these strategies (screening only, screening and surveillance, vs. none), irrespective of their effectiveness. Endoscopic screening and surveillance is expensive and can be risky. Therefore, it is imperative to establish the CE and acceptability about the risks and outcomes related to these practices to better inform expert recommendations and provider-patient decisions.

**Methods/Results:**

We propose a mixed methods study which will involve: (1) an analysis of secondary databases (VA and VA-Medicare linked datasets for 2004–09) to examine CE of endoscopic screening and surveillance in an observational study cohort (an estimated 680,000 patients with GERD; 25,000–30,000 with BE; and 3,000 with EA); (2) a structured electronic medical record (EMR) review on a national sample of patients using VA EMRs to verify all EA cases, identify cancer stage, cancer-targeted therapy, and validate the screening and surveillance endoscopy; and (3) qualitative in depth interviews with patients and providers to elicit preferences, norms, and behaviors to explain clinical contexts of these findings and address gaps arising from the CE study.

**Conclusion:**

This study will compare clinical strategies for detecting and monitoring BE, a pre-cancerous lesion. Additionally, by eliciting acceptability of these strategies for patients and providers, we will be able to propose effective and feasible strategies that are likely to be implemented in routine use. Findings will inform recommendations for clinical practice guidelines. Our innovative approach is consistent with the methodological standards of patient-centered outcomes research, and our findings will offer a significant contribution to the literature on cancer surveillance.

**Trial Registration:**

Not applicable

## Background

Esophageal adenocarcinoma (EA) is one of the fastest rising cancers in the United States [1-3]. The rise has been most dramatic among white men, with a five-fold increase between 1980 and 2005 [2,4]. EA is a highly fatal cancer with a median survival of less than one year following diagnosis [5]. Barrett’s esophagus (BE) is a detectable precursor lesion that offers a potential target for preventing EA. The risk of EA in individuals with BE is increased between 30 and 130-fold as compared to general population controls [6]. The annual incidence of EA in BE is estimated to be 0.5%. The onset of BE is silent, and therefore its exact time of onset is virtually never known. Elderly Caucasian men in developed countries have the highest risk of BE [7,8]. The presence of frequent chronic gastroesophageal reflux disease (GERD) symptoms has been consistently associated with an increased risk of BE as well as EA [9,10].

Current practice guidelines advocate endoscopic screening for adult individuals with chronic GERD symptoms [11]. Once BE is diagnosed, endoscopic surveillance is recommended [11-13]. The detection of early neoplastic changes (dysplasia or carcinoma in situ) prompts treatment with several potentially curative modalities of local ablation or esophageal resection [8,12-15]; the predicted benefit being reduction of EA incidence by treating BE with dysplasia, and improving EA outcomes.

Effectiveness of screening and surveillance endoscopy is highly dependent on the utilization of these procedures. However, the extent and patterns of endoscopic screening and surveillance in practice are unclear. Physician surveys indicate that more than 95% of gastroenterologists recommend BE surveillance [16]. There are no large-scale studies from community or healthcare system based settings documenting the use of screening or surveillance endoscopy among well-defined populations. Studies evaluating patients’ perceptions of cancer risk and acceptability of endoscopic surveillance for BE, and predictors of adherence to surveillance endoscopy demonstrate poor understanding of the available evidence and utilization that is driven by insurance type and availability of endoscopy services [17-22].

The comparative effectiveness (CE) of screening endoscopy (vs. no screening) in patients with upper GI symptoms, surveillance (vs. no surveillance) in BE patients, and CE of different surveillance strategies (frequency, intensity) is unclear due to a lack of evidence-based findings for GERD/BE populations at risk of EA. Existing studies examining the effectiveness of screening and surveillance endoscopy generally fall into one of two categories: (1) large retrospective population-based studies in which researchers examine EA patients’ charts for the presence of pre-diagnosis endoscopy and then form associations between endoscopy and stage of cancer at the time of diagnosis and treatment and survival following diagnosis; these studies have the uniform limitation of not examining the population at risk (GERD or BE), or (2) small studies of BE patients with relatively short follow up and very few EA cases. Though they provided information on the possible role of endoscopy in affecting the outcomes of EA, past studies have not addressed the frequency or yield in the population at risk. There have been no cohort studies addressing CE of screening or surveillance endoscopy in populations at risk. We propose to conduct such a CE study.

While there is limited knowledge about effectiveness of various EA screening and surveillance strategies, less is known about factors that shape physicians’ and patients’ decisions concerning such strategies. Patients’ decisions to participate in initial screening, repetitive surveillance, or treatment may be guided by their perceptions of cancer risk, their expected outcomes of participation, and by other affective responses to screening and surveillance experiences and norms. Risk perception is defined as the assessment of one’s personal risk for cancer diagnosis and cancer-related mortality. Most patients with BE incorrectly estimate their risk of EA. Shaheen et al. performed a study evaluating the risk perceptions for progression to EA among 118 patients with non-dysplastic BE undergoing surveillance endoscopy [22]. The study, which used a visual analog scale that was developed to factor low incidence risks [23], found that 68% of respondents over-estimated their 1-year risk of cancer (13.6% versus an actual risk of 0.05%) [22]. In contrast, a Dutch study of 192 patients with BE and no high-grade dysplasia found that participants underestimated their 1-year risk of cancer [21]. Taken together these data underscore the notion that patients with BE may not pursue screening and surveillance behaviors based on inaccurate perceptions of cancer risk. No data is available to accurately estimate physicians’ risk perceptions or describe how physicians and patients discuss and reach consensus on the benefits and risks of endoscopic surveillance.

Physicians often over-appreciate the benefits of diagnosing BE. It has been suggested that physicians largely do not balance the real social harms and costs of diagnosing BE against the potential benefits of cancer prevention. A diagnosis of BE alone was found to double health and life insurance premiums across a wide range of surveyed national insurance companies [24]. Sparse data exists to inform physicians’ outcome expectancies for surveillance and ablative therapy. Some clinical and decision science experts have even advocated against surveillance (while still supporting screening) due to the significant limitations, uncertain benefits, and potential costs [25-27]. Better evidence is needed to clarify how physicians perceive the quantitative and qualitative benefits/risks of surveillance and therapeutic endoscopy and how such information is communicated to patients to arrive at informed decisions.

In light of the many gaps in knowledge regarding the extent of utilization and perceptions of endoscopic screening and surveillance, the CE of various strategies, and the patient and provider factors that shape decisions to adopt current and future practice guidelines, we propose to conduct a study with the following specific aims. In this article, *screening* endoscopy is defined as endoscopy in patients with no known BE or EA, while *surveillance* is endoscopy in patients with known BE.

### Specific aims

#### Comparative effectiveness aims

To compare the risk (detection rate) and outcomes (stage, treatment, survival) of EA among patients undergoing different intensities of screening and surveillance endoscopy. We hypothesize that screening (vs. none) and surveillance endoscopy (once every 2 years, or once every 3 years vs. none) will increase the likelihood of patients: being diagnosed with early stage EA; receiving treatments for EA; and having lower EA-specific mortality.

To identify predictors of desired outcomes of EA (low incidence and low EA mortality). Potential predictors include demographic features (e.g. age), GERD features (e.g. duration), interventions (e.g. PPI, fundoplication, ablation), and other BE risk factors (e.g. obesity, smoking).

#### Qualitative aim

To elicit physicians’ and patients’ risk perceptions, outcome expectancies, and affective responses to endoscopic screening and surveillance strategies and ablative therapy. Using an integrated model of decision making and health behavior, we will describe how these various perceptions potentially effect adoption and implementation of surveillance endoscopy.

## Methods

### Design overview

To address these aims, we propose a study with a mixed methods approach where we will (1) use secondary databases (VA and VA-Medicare linked datasets for 2004–09) to examine CE of screening and surveillance in an observational study cohort (an estimated 680,000 patients with GERD, 25,000–30,000 with BE, and 3,000 with EA); (2) conduct a detailed structured electronic medical record (EMR) review on a national sample of patients using VA electronic medical records to verify all EA cases, identify cancer stage, cancer-targeted therapy, and validate screening and surveillance endoscopy; and (3) generate qualitative data from in-depth interviews on patient and provider preferences, rationale, and behavioral utilities to explain some of the findings from the cohort study and inform the recommendations resulting from this research. This human subjects study was approved by the Institutional Review Board of Baylor College of Medicine (Comparative Effectiveness of Screening and Surveillance Endoscopy, protocol H-27619).

### Comparative effectiveness aim

Data for this aim will come from the Austin Information Technology Center (AITC). The AITC houses several administrative datasets for the VA, including: Medical SAS Inpatient and Outpatient files, Decision Support System (DSS) files, and the Vital Status File. The SAS Inpatient and Outpatient files provide detailed patient demographic characteristics, such as date of birth and race/ethnicity, as well as procedure (CPT codes) and diagnosis (ICD-9 codes) codes from inpatient and outpatient visits. The DSS includes select laboratory test results and pharmacy information. The Vital Status File contains patients’ date of death. VA-Medicare is a linked file of all Medicare claims made by VA users. This file will be used to ascertain additional information for Medicare eligible (mostly 65 years and older) patients who may have co-utilized services outside the VA. We will also perform structured chart abstraction of the VA EMR on all patients with EA in the VA nationwide and a random sample of patients with screening and surveillance endoscopy with GERD and/or BE. We will access the EMRs using Compensation and Pension Record Interchange (CAPRI), a VA application that provides access to the EMR found in the Computerized Patient Record System (CPRS) at any VA facility nationwide.

#### Participants

Patient participants include approximately 680,000 veterans with GERD, 25,000-30,000 with BE, and 3,000 with EA. These patients will all be veterans enrolled in the VA healthcare system. A *waiver of consent and authorization* has been approved by the local IRB for this aim of the protocol.

#### Inclusion criteria

We will include patients between 18 and 90 yrs old *with GERD* and/or BE. Patients with GERD will be identified in the outpatient VA files during 2003–09 with a one inpatient or two outpatient ICD-9 codes for GERD. Patients *with BE* will be defined by the presence of ICD-CM-9 code 530.85 combined with at least one EGD test (CPT codes 43200–43259, excluding 43246) within 12 months after the BE code date.

#### Exclusion criteria

In order to identify candidates for surveillance, we will exclude those with any gastric duodenal or esophageal cancers, abdominal surgery, decompensated liver disease, feeding tube, any metastatic cancer, or chemotherapy. These patients will be identified by related ICD-9 codes within 5 years preceding BE index date. To maximize the likelihood of having new GERD diagnosis, we will also exclude patients with previous GERD diagnosis preceding GERD index date during the study period.

#### Study variables

Exposure: endoscopy procedures recorded in the study cohort will be classified as screening, surveillance, or diagnostic endoscopy. We define that (1) Screening endoscopy is the first non-diagnostic endoscopy recorded after the index date for GERD; (2) Surveillance endoscopy is any non-diagnostic endoscopy performed in patients with BE (after the screening endoscopy where BE is diagnosed); and (3) Diagnostic endoscopy is any endoscopy performed in patients with “alarm” features in the 1 year preceding endoscopy date; these include low hemoglobin and anemia (Hgb <12 g/dl, and <8.5 g/dl, respectively), dysphagia, and GI bleeding (hemetemsis, melena, hematochezia), and any of the exclusion criteria mentioned above that may develop after the GERD index date.

Follow up: All patients in the cohort will have follow up till death, development of EA, or end of study period in 09/30/2011.

Outcomes: 1) EA diagnosis based on a structured EMR review for diagnosis and treatment related to EA where EA is defined based on endoscopic and histological criteria; 2) EA stage as determined from tumor board or results of diagnostic tests; 3) any receipt of EA treatment; 4) EA related death.

Potential Confounders and Effect Modifiers: Confounding factors, observed and unobserved, can affect each process over the follow-up periods and may generate biases that affect the main study estimates. The below variables will be considered as potential confounders.

Patient Factors: Socio-demographic factors, including age, gender, race, ethnicity, marital status, and socioeconomic status. VA priority levels will be used as a surrogate for socioeconomic status. Clinical characteristics will include medical and psychiatric comorbidities. We will also evaluate filled prescriptions for medications (e.g. PPI or H2-receptor antagonists) including measures of adherence (medication-ownership ratio) and persistence (length of therapy, fill-refill ratio, and discontinuation rate) [28-30], BE risk factors (e.g., BMI, smoking), BE treatment (photodynamic therapy, mucosal resection, radiofrequency ablation, or cryoablation), and the presence of dysplasia.

Non-Patient Factors: We will identify the VA facility for each patient following the date of GERD or BE diagnosis. Facility factors will include urban or rural setting, regions, academic affiliation, overall patient load, and GERD and BE load. We will examine provider type by using clinic stop codes for primary care providers and gastroenterologists.

### Qualitative aim

#### Participants

We will conduct one-on-one in-depth interviews with a targeted sample of up to 70 individuals (45 patients and 25 physicians) who meet the specified inclusion criteria. One-on-one interviews are useful for exploring individuals’ opinions, and are designed to render stories and provide information depth [31]. We chose the sample size of 70 because it is expected to generate sufficient data to perform a qualitative analysis of patients’ and physicians’ beliefs and corresponding decisions regarding screening, surveillance, and treatment for GERD and various degrees of BE (i.e. BE with high grade dysplasia, BE with low-grade dysplasia, BE without dysplasia). All interviewees will provide written consent prior to participation and all interviews will be recorded, transcribed, and analyzed for content.

We will apply a stratified purposeful sampling technique to recruit patients who meet the criteria for three categories of interest, including: 1) patients with GERD who are scheduled to undergo their first ever endoscopy (see Additional file 1: Appendix I), 2) patients who have been diagnosed with BE (with or without dysplasia), and 3) patients who have been diagnosed with BE and have undergone at least one ablation (see Additional file 1 II for the later two categories). The stratified purposeful sampling strategy allows for the sampling of subgroups and facilitates comparisons between the subgroups [32]. We will review EMRs from the Michael E. DeBakey VA Medical Center (MEDVAMC) to identify patients treated at the MEDVAMC gastroenterology specialty clinic, and we will identify physicians using the list of practicing gastroenterologists in three local GI practices, including one VA hospital.

Patient interviews will elicit information about etiological understandings of their condition (GERD or BE), reactions to their GERD or BE diagnosis, beliefs and expectations about endoscopy, perceptions of risk of developing EA, barriers and facilitators of undergoing endoscopy, and beliefs and expectations about ablative therapy, if relevant. Interview guides will be tailored for the three categories of interest described above (See Appendices I and II). We will also conduct individual, in-depth interviews with up to 25 gastroenterologists to obtain information about their experiences with screening and surveillance endoscopy in patients with GERD and/or BE (see Additional file 1: Appendix II). Interviews will elicit information about physicians’ screening, surveillance, and ablation practices, their beliefs about EA risk, perceptions of effectiveness of screening and surveillance endoscopy, and their perceptions of patients’ beliefs. We will conclude sampling at the point of thematic saturation, or when no new insights are gleaned from the interviews.

### Analysis

#### Power and sample size

For the CE aims, we conservatively expect 500,000 patients with GERD and 20,000 BE patients in our study cohort. Using the estimated number of BE patients, an average annual EA rate in BE of 0.5%, and an average follow up for BE patients of 5 years, we expect to find at least 500 EA cases in the context of an existing BE. We assume based on preliminary work that approximately 33% will have a surveillance endoscopy every 2 years, 33% have a surveillance endoscopy every 3 years, and 33% will have no endoscopy. The favorable outcomes in the 2-year surveillance group will be early stage EA diagnosis for 30% of cases, EA curative treatment in 25%, and 3-year survival in 50%. Since there are 3 comparisons we set alpha = 0.025 and power = 0.80. Therefore, with 166 patients in each group, we will be able to detect differences of (50% vs. 34%), (30% vs. 15%), and (25% vs. 12%) in any of the outcomes between the every 2 year surveillance and no surveillance groups.

Sample size estimates for the qualitative aim will be based on established principles of thematic saturation. We will conclude sampling for each subgroup at the point of thematic saturation for each patient group [33]. We will assume thematic saturation when all coders agree that three consecutive transcripts from each patient group render no new insights. We will also establish thematic saturation for physicians as a distinct group and continue recruitment and coding until all coders agree that three consecutive transcripts are saturated across all major themes.

#### Data analysis for the CE aims

We will calculate the cumulative and yearly EA incidence rates overall and in groups stratified by disease status (GERD with or without BE) and endoscopy status (screening, surveillance, none); we will also calculate incidence rate ratios (and 95% CI). The main comparison groups are (screening vs. no screening) and (surveillance every 2 years or surveillance every 3 years vs. no surveillance). Similar comparisons will be made for the proportions of patients with early EA stage and whether they received EA treatment. We will examine patient and non-patient predictors that may serve as clinically or epidemiologically useful predictors of the study outcomes. For EA stage and treatment receipt, we will use logistic regression models. For survival, we will use multivariable hierarchical Cox proportional hazards modeling to assess the effect of screening or surveillance on mortality risk while adjusting for patient and non patient features.

We will use propensity score matching to adjust for the non-random probability of receiving endoscopic screening or surveillance. We will use logistic regression analyses to determine the probability (propensity score) of receiving screening endoscopy based on patient and non-patient factors detailed above, and match the subjects receiving screening with those who did not receive screening based on the propensity score.

#### Analysis for the qualitative aim

We will analyze in-depth interview data using principles of framework analysis, which allows for the inclusion of existing concepts as well as emergent themes [34]. The advantage of framework analysis is that it provides a clear and systematic approach to managing and analyzing large quantities of complex data. This approach allows themes to develop both from the research questions and from the narratives of research participants. In this way, our analysis will apply both inductive and deductive methods. There are five stages in framework analysis: familiarization, identification of a thematic framework, indexing (coding), charting themes, and mapping and interpretation (See Additional file 1: Appendix IV for coding framework).

Two independent coders with experience in framework analysis will independently create codes and index the data using the software package Atlas. ti 5.0. We will use the constant comparative method of data interpretation, which involves making comparisons at every stage of the analysis [35]. For instance, we will compare earlier data with later data, interviews conducted by different interviewers, and a priori themes with emergent themes. Our coding procedure will involve several steps. First, coders will become familiarized with all the data by reading, rereading, and summarizing each transcript. Each transcript will be supplemented with observational notes, which will allow us to gain an overall impression of the interviews and focus group, and to identify “key ideas and recurrent themes” about BE and endoscopy [34]. Based on these insights, coders will begin creating an overall thematic framework, incorporating both emergent and a priori themes from our conceptual model. The research team will discuss this framework and develop a coding scheme. In order to reduce bias and ensure inter-coder reliability and the utility of the codes, two members of the research team will independently code 6 transcripts [36]. We will identify inconsistencies and emergent ideas, and modify the coding manual accordingly. Two coders will then systematically apply the thematic framework to all transcripts. During this indexing procedure, coders will add and modify the coding scheme as necessary. We will chart commonalities and divergent themes within and among all participant subcategories. We will merge coded transcripts in Atlas. ti and the team will review the data. Disagreements about coding decisions will be resolved through group consensus [36]. Finally, we will use the thematic framework to chart the themes across focus groups to identify saliency and compare and contrast participants’ accounts to identify meaningful associations and patterns in the data (mapping and interpretation). Once the data are organized and thematically related, we will identify a central category that best summarizes the relationships observed in the data.

### Triangulation of data

We expect wide variations in the utilization of endoscopic screening and surveillance with predominant findings of underutilization as well as some overutilization likely driven by small-area variations in the supply of endoscopic services. In additional to descriptive results on a healthcare system wide basis, quantitative analysis may provide needed evidence regarding the benefits of screening on outcomes of interest (mortality, stage at diagnosis, increased use of surveillance following screening, etc), and key data to inform outcome expectancies of surveillance endoscopy (i.e. which surveillance interval is likely to improve mortality and recognition of dysplasia progression). We also expect that several quantifiable and potentially modifiable patient, provider, and facility factors will be predictive of receipt (or non receipt) of endoscopy.

As in most observational studies, we expect that these quantitative analyses will explain only a small proportion of the variations in practice. Our implicit chart EMR review in selected groups of patients will help maximize the collection of variables and thus increase the explained portion of variation. However, we believe that most of the variation cannot be explained by physiological factors that can be quantified by the databases, and therefore our qualitative aim will offer insight into how physicians’ perceptions and biases shape their decisions regarding endoscopy, as well as how patients’ perceptions of cancer risk, their expected outcomes of participation, and by other affective responses to screening and surveillance guide their decisions to participate in screening and surveillance. We expect that in certain subgroups the CE of endoscopic screening and especially surveillance will be favorable, but as described above, widely misutilized. As is common in qualitative research, our findings are most likely to generate themes related arising from responses to our interview guides. Interpretation of thematic data is valuable but often highly contextual. To improve the generalizability of our data, we will attempt to place our qualitative themes within the framework of contemporary psychological models of decision making and health behavior [37,38]. Within this framework, our qualitative findings will help to inform where key gaps remain in the decision making process to pursue screening and surveillance. Additional analyses can be planned to address these gaps, at least partially, if the data is available.

The next logical step is to implement strategies to correct the utilization of endoscopic screening and surveillance. The findings of the qualitative aim will be crucial in designing these interventions, particularly if most the practice variations cannot be attributed to patient related clinical factors. Informed by the findings of our quantitative results, additional qualitative interviews with patients and physicians will explore the comparative acceptability of alternative screening and surveillance protocols. These findings will provide insights into the adoption and implementation of revised guidelines for endoscopic screening and surveillance.

## Results and discussion

A tremendous amount of resources are spent each year on performing endoscopic procedures to screen and survey for BE to prevent EA; however, there is limited knowledge about the effectiveness of various EA screening and surveillance strategies, and even less is known about factors that shape physicians’ and patients’ decisions concerning the adoption and implementation of these strategies.

The aims of this CE study are to generate a better understanding of particular patterns of endoscopy (frequency and risk factors) that are associated with tangible benefits in terms of reducing the incidence or mortality from EA. We will provide insight into physicians’ decisions to perform screening, surveillance, and treatment procedures, and adherence (or not) to recommended guidelines. Additionally, we will gain a better understanding of affective and structural factors that shape patients’ risk perceptions and decisions to adhere to cancer screening and surveillance guidelines. Ultimately, our findings have the potential to inform evidence-based guidelines and clinical practice.

CE research is not a new concept and there is criticism regarding its ability to impact routine clinical care [39]. The American Recovery and Reinvestment Act (2009) and Patient Protection and Affordable Care Act (2010) provided substantial new investments in CE research. As a response, the Methodology Committee of the Patient-Centered Outcomes Research Institute—itself funded by the Affordable Care Act, created a set of methodological standards for conducting “patient-centered” CE research [40]. The four standards include 1) prioritizing research questions, 2) using appropriate study designs and analyses, 3) incorporating patient perspectives, and 4) fostering efficient dissemination and implementation of results. Our proposed CE study of screening and surveillance endoscopy is likely to inform policy and practice because the study aligns with the four standards articulated by the PCORI committee. First, we have prioritized our research aims on the key clinical questions facing physicians and patients regarding endoscopic screening and surveillance. As part of the study, we begin with qualitative interviews with patients and physicians to clarify the knowledge and perception gaps that hamper good decision making. The results of these interviews will shape the quantitative analyses. Second, we use appropriate study designs and analytical approaches to avoid biases and errors common to observational studies. We use a structured chart review to improve the validity and reliability of our outcome definitions. Furthermore, we use specialized analytical techniques (i.e. propensity score matching) to reduce bias in our analytical models [41]. Third, patients are engaged throughout the research process to inform the identification of key clinical questions, interpretation of research findings, and the potential adoption and implementation of guideline recommendations arising from study conclusions. In particular, we will frame the input received from patients within models of decision making and health behavior as they relate to screening and surveillance endoscopy. It is through this modeling that key clinical questions arise and research findings can be easily interpreted into patient-physician decisions. Finally, our study fosters efficient adoption and implementation of results through the involvement of practicing physicians throughout the study process. As with patients, we interview physicians to inform our key clinical questions and to interpret the findings within everyday clinical contexts. The involvement of patients and physicians throughout the study dramatically improves the real-world relevance of the study findings and enhances their likelihood for adoption and implementation in routine care.

## Conclusions

The proposed CE study is consistent with the Patient Centered Outcomes Research Institute (PCORI) methodological standards for CE research. The current study is consistent with the principle of patient-centeredness by using a mixed-methods approach that includes in-depth qualitative interviews with physicians and patients who are intimately involved with the questions of screening and surveillance endoscopy. Qualitative methods are used to improve the clinical relevance and patient-centeredness of the research questions and findings. The study includes the largest national sample of patients undergoing screening/surveillance endoscopy and uses appropriate study designs and analyses. In summary, the proposed CE study has the real potential to impact policy and practice through its conformity with the PCORI methodological standards for patient-centered CE research.

## Competing interests

There are no financial or non-financial competing interests for any of the authors.

## Authors’ contributions

JRK: Participated in the study design and drafting of the protocol. JA: Participated in the design and drafting of the qualitative aims of the protocol. JC: Participated in the design and drafting of the data analysis plan in the protocol. PR: Participated in the design and drafting of the data analysis plan in the protocol. ZD: Participated in drafting the data analysis plan. RLS: Conceived of the integration of the qualitative and quantitative aims and drafting of the protocol. MHL: Participated in the qualitative analysis section of the protocol and the overall coordination of the project. ADN: Conceived of the design of the qualitative aims and participated in drafting the protocol. HES: The principal investigator of the grant proposal: conceived of the study, obtained funding, and participated in designing and drafting the protocol. All authors read and approved the final manuscript.

## Pre-publication history

The pre-publication history for this paper can be accessed here:

http://www.biomedcentral.com/1472-6963/12/288/prepub

## Supplementary Material

Additional file 1 I. Patient Interview Guide: Pre-diagnosis II. Patient Interview Guide: Post-diagnosis (no dysplasia, low- and high-grade dysplasia) III. Physician Interview Guide IV. Framework Analysis Coding Key (Patient Interviews). Click here for file

## References

[B1] Blot WJ McLaughlin JK The changing epidemiology of esophageal cancer Semin Oncol 1999 26 2 8 10566604

[B2] Devesa SS Blot WJ Fraumeni JF Jr Changing patterns in the incidence of esophageal and gastric carcinoma in the United States Cancer 1998 83 2049 2053 10.1002/(SICI)1097-0142(19981115)83:10<2049::AID-CNCR1>3.0.CO;2-2 9827707

[B3] Sampliner RE Barrett's Esophagus, a complication of GERD Current Treatment Options in Gastroenterology 2002 5 45 50 10.1007/s11938-002-0006-1 11792237

[B4] El-Serag HB The epidemic of esophageal adenocarcinoma Gastroenterol Clin North Am 2002 31 421 440 10.1016/S0889-8553(02)00016-X 12134611

[B5] Eloubeidi MA Mason AC Desmond RA El-Serag HB Temporal trends (1973-1997) in survival of patients with esophageal adenocarcinoma in the United States: a glimmer of hope? Am J Gastroenterol 2003 98 1627 1633 12873590 10.1111/j.1572-0241.2003.07454.x

[B6] El-Serag HB Mason AC Petersen N Key CR Epidemiological differences between adenocarcinoma of the oesophagus and adenocarcinoma of the gastric cardia in the USA Gut 2002 50 368 372 10.1136/gut.50.3.368 11839716 PMC1773122

[B7] Cameron AJ Epidemiology of columnar-lined esophagus and adenocarcinoma Gastroenterol Clin North Am 1997 26 487 494 10.1016/S0889-8553(05)70308-3 9309399

[B8] Sharma P McQuaid K Dent J A critical review of the diagnosis and management of Barrett's oesophagus: the AGA Chicago workshop Gastroenterol 2004 127 310 330 10.1053/j.gastro.2004.04.010 15236196

[B9] Eisen GM Sandler RS Murray S Gottfried M The relationship between gastroesophageal reflux disease and its complications with Barrett's Esophagus Am J Gastroenterol 1997 92 27 31 8995932

[B10] Lieberman DA Oehlke M Helfand M GORGE Consortium. Risk factors for Barrett's Esophagus in community-based practice Am J Gastroenterol 1997 92 1293 1297 9260792

[B11] Sampliner RE Updated guidelines for the diagnosis, surveillance, and therapy of Barrett's Esophagus Am J Gastroenterol 2002 97 1888 1895 10.1111/j.1572-0241.2002.05910.x 12190150

[B12] Sampliner RE Practice guidelines on the diagnosis, surveillance, and therapy of Barrett's Esophagus. The Practice Parameters Committee of the American College of Gastroenterology Am J Gastroenterol 1998 93 1028 1032 10.1111/j.1572-0241.1998.00362.x 9672324

[B13] Spechler SJ Clinical practice. Barrett's Esophagus New England Journal of Medicine 2002 346 836 842 10.1056/NEJMcp012118 11893796

[B14] Sampliner RE Prevention of adenocarcinoma by reversing Barrett's Esophagus with mucosal ablation World J Surg 2003 27 1026 1029 10.1007/s00268-003-7056-y 12917762

[B15] Spechler S Managing Barrett's Oesophagus. Br Med J 2003 326 892 894 10.1136/bmj.326.7395.892 PMC1125811 12714452

[B16] Gross CP Canto MI Hixson J Powe NR Management of Barrett's Esophagus: a national study of practice patterns and their cost implications Am J Gastroenterol 1999 94 3440 3447 10.1111/j.1572-0241.1999.01606.x 10606300

[B17] Cooper GS Kou TD Chak A Receipt of previous diagnoses and endoscopy and outcome from esophageal adenocarcinoma: a population-based study with temporal trends Am J Gastroenterol 2009 104 1356 1362 10.1038/ajg.2009.159 19491849

[B18] Cooper SC El-agib A Dar S Endoscopic surveillance for Barrett's oesophagus: the patients' perspective Eur J Gastroenterol Hepatol 2009 21 850 854 10.1097/MEG.0b013e328318ed2d 19598328

[B19] Crockett SD Lippmann QK Dellon ES Shaheen NJ Health-related quality of life in patients with Barrett's esophagus: a systematic review Clin Gastroenterol Hepatol 2009 7 613 623 10.1016/j.cgh.2009.02.024 19281858 PMC2693470

[B20] Kruijshaar ME Kerkhof M Siersema PD Steyerberg EW Homs MY Essink-Bot ML The burden of upper gastrointestinal endoscopy in patients with Barrett's esophagus Endoscopy 2006 38 873 878 10.1055/s-2006-944613 17019759

[B21] Kruijshaar ME Siersema PD Janssens AC Kerkhof M Steyerberg EW Essink-Bot ML Patients with Barrett's esophagus perceive their risk of developing esophageal adenocarcinoma as low Gastrointest Endosc 2007 65 26 30 10.1016/j.gie.2006.05.030 17185076

[B22] Shaheen NJ Green B Medapalli RK The perception of cancer risk in patients with prevalent Barrett's esophagus enrolled in an endoscopic surveillance program Gastroenterology 2005 129 429 436 16083700 10.1016/j.gastro.2005.05.055

[B23] Woloshin S Schwartz LM Byram S Fischhoff B Welch HG A new scale for assessing perceptions of chance: a validation study Med Decis Making 2000 20 298 307 10.1177/0272989X0002000306 10929852

[B24] Shaheen NJ Dulai GS Ascher B Mitchell KL Schmitz SM Effect of a new diagnosis of Barrett's Esophagus on insurance status Am J Gastroenterol 2005 100 577 580 10.1111/j.1572-0241.2005.41422.x 15743354

[B25] Barritt AS Shaheen NJ Should patients with Barrett's Oesophagus be kept under surveillance? The case against Best Practices Research Clinical Gastroenterology 2008 22 741 750 10.1016/j.bpg.2007.12.005 18656827

[B26] Inadomi JM Sampliner RE Lagergren J Lieberman D Fendrick AM Vakil N Screening and surveillance for Barrett's Esophagus in high-risk groups: a cost-utility analysis Ann Intern Med 2003 138 176 186 12558356 10.7326/0003-4819-138-3-200302040-00009

[B27] Inadomi JM Somsouk M Madanick RD Thomas JP Shaheen NJ A cost-utility analysis of ablative therapy for Barrett's Esophagus Gastroenterology 2009 136 2101 2114 10.1053/j.gastro.2009.02.062 19272389 PMC2693449

[B28] Cramer JA Roy A Burrell A Medication compliance and persistence: Terminology and definitions Value in Health 2008 11 44 47 10.1111/j.1524-4733.2007.00213.x 18237359

[B29] Peterson AM Nau DP Cramer JA Benner J Gwadry-Sridhar F Nichol M A checklist for medication compliance and persistence studies using retrospective databases Value in Health 2007 10 3 12 10.1111/j.1524-4733.2006.00139.x 17261111

[B30] Steiner JF Prochazka AV The assessment of refill compliance using pharmacy records: methods, validity, and applications J Clin Epidemiol 1997 50 105 116 10.1016/S0895-4356(96)00268-5 9048695

[B31] Miller WL Crabtree BF Crabtree BF, Miller WL Depth interviewing Doing QualitativeResearch 1999 2 Thousand Oaks: Sage 89 108

[B32] Kuzel AJ Crabtree BF, Miller WL Sampling in qualitative inquiry Doing Qualitative Research 1999 2 Thousand Oaks: Sage 33 46

[B33] Morse J The significance of saturation Qual Health Res 1995 5 147 149 10.1177/104973239500500201

[B34] Ritchie J Spencer L Bryman A, Burgess RG Qualitative data analysis for applied policy research Analyzing Qualitative Data 1994 New York: Routledge 173 94

[B35] Glaser BG Strauss AL The Discovery of Grounded Theory: Strategies for Qualitative Research 1967 Chicago: Aldine

[B36] Waitzkin H The Politics of Medical Encounters: How Patients and Doctors Deal with Social Problems 1991 New Haven: Yale University Press

[B37] Reyna VF A theory of medical decision making and health: fuzzy trace theory Med Decis Making 2008 28 850 865 10.1177/0272989X08327066 19015287 PMC2617718

[B38] Fishbein M A reasoned action approach to health promotion Med Decis Making 2008 28 834 844 10.1177/0272989X08326092 19015289 PMC2603050

[B39] Naik AD Petersen LA The neglected purpose of comparative-effectiveness research N Engl J Med 2009 360 1929 1931 10.1056/NEJMp0902195 19420362 PMC2868830

[B40] Methodology Committee of the PCORI Methodological standards and patient-centeredness in comparative effectiveness research J Am Med Assoc 2012 307 1636 1640 10.1001/jama.2012.466 22511692

[B41] Concato J Is it time for medicine-based evidence? J Am Med Assoc 2012 307 1641 1643 10.1001/jama.2012.482 22511693

