# Supplementary material for: Patient-centered, comparative effectiveness of esophageal cancer screening: protocol for a comparative effectiveness research study to inform guidelines for evidence-based approach to screening and surveillance endoscopy
Source: BMC Health Serv Res. 2012 Aug 28;12:288. doi: 10.1186/1472-6963-12-288 (PMC3508612; doi:10.1186/1472-6963-12-288)
Supplement: Additional file 1 — I. Patient Interview Guide: Pre-diagnosis II. Patient Interview Guide: Post-diagnosis (no dysplasia, low- and high-grade dysplasia) III. Physician Interview Guide IV. Framework Analysis Coding Key (Patient Interviews). [file 1472-6963-12-288-S1.doc]

**Appendices**

I. Patient Interview Guide: Pre-diagnosis

II. Patient Interview Guide: Post-diagnosis (no dysplasia, low- and high-grade dysplasia)

III. Physician Interview Guide

IV. Framework Analysis Coding Key (Patient Interviews)

Pre-Diagnosis Group

Brief History

Gender: _____________________

DOB:________________________

When were you diagnosed with GERD? __________________

How many endoscopies have you had in all of your life? ____________

How many endoscopies have you had since you were diagnosed with GERD? _________

Have they all been at the VA? _________________________________

If no, where ________________________________________

When is your next scheduled endoscopy? _______________________________

*General Health*

Tell me about your health? What are your main concerns right now?

*Symptoms*

Tell me about the problems/issues you are experiencing related to your stomach?

How do these problems/issues feel?

Tell me about any pain or discomfort?

What other problems does it cause? Trouble swallowing or eating?

How long have you had these problems/issues?

Are there certain things that seem to trigger your problems/issues?

How have your problems/issues changed over time?

Gotten better or worse?

What do you think is causing your problems/issues?

What does this mean to you?

What do you think could happen because of your problems/issues?

Have any of your friends or family members had these same problems/issues? What have they said about it?

Did they give advice about managing problems/issues?

Did they recommend you do anything else – like see a doctor/get Rx?

Why did you see a doctor for these problems/issues?

Do you have a regular source of care for these problems/issues?

PCP? Referred to specialist?

Tell me about your experiences with the doctor related to these problems/issues?

What does your doctor think is the cause of these problems/issues?

After talking to the doctor, what do you think about these problems/issues?

What is the cause of your problems/issues?

Have you been diagnosed with a specific condition?

*Symptom Management*

What have you done to manage your problems/issues?

What did you try on your own? (i.e. Diet changes like no more fried or spicy foods, no caffeine, no late eating, smaller more frequent meals, decrease smoking, elevate head of your bed, stop or start exercising, lose weight)

Why did you try that?

Was that helpful? Tell me about that.

Did any other problems arise when you tried these things?

What did you do to manage these new problems?

What has your doctor recommended you try? Medications/treatments?

What have you tried?

Tell me about that? Did it work?

Did any other problems arise when you tried these things?

Did you discuss these problems with your doctor?

What have you done to manage these new problems?

*Endoscopy*

I understand that your doctor has recommended that you have an endoscopy.

What *is* an endoscopy?

Where did you get this information? From your doctor? From a friend? Seek info?

Why does your doctor want you to have this procedure?

How did the recommendation make you feel? (nervous/relieved?)

Will you tell me more about that? Why did you feel ______?

Did the doctor tell you about things the endoscopy might find?

Probe: understanding of condition/screening for BE/link between BE-EA

After your appointment did you discuss what the doctor told you with anyone?

Who did you talk to? (significant other, family, friends)

What did you talk with them about?

i.e. Talk about the procedure or their concerns?

What did they say or recommend?

Did they want you to have the endoscopy?

Were they worried about the possible outcomes?

Did they discuss their past experiences with you?

After you spoke to ______ did you feel differently about having an endoscopy?

How so? Tell me about that.

Are you planning on having the endoscopy?

Tell me about that.

If yes: Why are you choosing to have the endoscopy?

Probe: desire to get a diagnosis, to rule out BE, improve problems/issues?

Are there any other personal reasons you intend to have it?

If: “my doctor told me to”

Probe: Tell me about that. Trust your doctor?

Do you completely trust your doctor’s decisions about which medical treatments are best? Why or why not?

If no: Why are you choosing not to have the endoscopy

Do you have concerns specifically about the procedure?

Or possible outcomes of the screening?

If they say it wasn’t that important to them (low benefit)

Probe: why is it not important?

Do you think that other people with the same problems as yours are having endoscopies?

Why do you think they are? Or are not?

Does this shape your decision?

In what way?

If your spouse’s/loved one’s physician recommended that they have an endoscopy screening would you want them to do it?

Why?

If this is a different answer than they gave for themselves then we would probe why they would feel it important for significant other and not themselves.

So you say you plan to have the endoscopy. Will you describe what you expect to happen on that day?

*Tease out feelings here. They will talk about different steps of this process. For each step:*

Will you say more about how you feel about _______?

ALT: So you say you’re not going to have the endoscopy. Can you tell me what you would expect to happen if you *did* have the endoscopy? What would happen on that day?

What, do you think, are the benefits to undergoing an endoscopy?

Continue to probe with “anything else, or can you think of any other benefits”

Did the doctor tell you about benefits of the endoscopy?

What do you think are the drawbacks to getting the endoscopy?

Continue to probe with “anything else, or can you think of any other drawbacks”

Did the doctor talk about risks of the endoscopy?

How did that information make you feel?

For you is your benefits list or your drawbacks list stronger?

Why?

Did these things help you make your decision to undergo the endoscopy?

In what way?

In terms of your emotions, how do you think you’ll feel after the endoscopy is over?

Will you tell me more about that?

Why do you think you’ll feel that way?

What do you expect to result from the endoscopy?

What will the procedure tell the doctor?

Probe to elicit information about health outcomes (i.e. I could get a diagnosis, I could rule out BE, I could improve problems/issues.)

*For any outcome they list, probe to elicit feelings* *and what that would mean to them.*

Probe: How does that make you feel?

How do you think the EGD might change your understanding of what is going on with your body?

Please tell me more about that?

How does that make you feel?

What do you think could happen if you do not have the endoscopy?

Probe: In terms of your health, what could happen?

How does that make you feel?

Mental Model

Is this experience with your problems/issues and testing similar to any other health problem you have experienced before?

Can you compare this experience to anything (like a health issue or any other event)?

Can you tell me more about that?

So now that you have thought through all of these details about your problems/issues and the endoscopy. Can you tell me generally what you make of your problems/issues and how you feel about the endoscopy?

Do you think how you feel about your problems/issues will change after your endoscopy?

Is there anything else related to your problems/issues or the endoscopy that we haven’t covered? Anything you think is important?

**Post-BE Diagnosis Group**

**Brief History**

Gender: _____________________

DOB:________________________

When were you diagnosed with BE? __________________

How many endoscopies have you had in all of your life? ____________

How many endoscopies have you had since you were diagnosed with BE? _________

Have they all been at the VA? _________________________________

If no, where ________________________________________

How often are you currently having the endoscopies? _______________________________

ASK THESE QUESTIONS TO ALL PARTICIPANTS

***General Health***

Tell me about your health? What are your main concerns right now?

***Symptoms***

Tell me about the problems/issues you are having with your stomach?

How do these problems feel?

Tell me about any pain or discomfort?

What other problems does it cause? i.e. Trouble swallowing or eating, disrupts sleep?

How long have you had these problems?

Are there certain things that seem to trigger your stomach problems?

How have these problems changed over time?

Gotten better or worse?

What do you think is causing your stomach problems?

What does this mean to you?

What do you think could happen because of your symptoms?

Have any of your friends or family members had these same stomach problems?

What have they said about it?

Did they give advice about how to take care of these problems?

Did they recommend you do anything else – like see a doctor/get Rx?

Why did you see a doctor for these problems?

Do you see your regular doctor for your stomach problems or a stomach specialist?

Tell me about your experiences with the doctor related to your stomach problems?

What does your doctor think is causing of these problems?

After talking to the doctor, what do you think about these problems?

What do you think is the cause of your stomach problems?

What condition have you been diagnosed with?

***Symptom Management***

What have you done to take care of your stomach problems?

What did you try on your own? (i.e. Diet changes like no more fried or spicy foods, talking to a pharmacist, buying medicines at the store, no caffeine, no late eating, smaller more frequent meals, decrease smoking, elevate head of your bed, stop or start exercising, lose weight)

Why did you try that?

Was that helpful? Tell me about that.

Did any other problems arise when you tried these things?

What did you do to manage these new problems?

What has your doctor recommended you try? Prescription medications/treatments?

Of those things what have you tried?

Tell me about that? Did it work?

Did any other problems arise when you tried these things?

Did you discuss these problems with your doctor?

What have you done to manage these new problems?

***Diagnosis***

Earlier you said you were diagnosed with ____________

Can you tell me about this diagnosis?

What does it mean?

What did the doctor say about _____?

When you were first diagnosed with _____ how did it make you feel? What were your first thoughts?

What were your main concerns when the doctor told you that you had _____?

How has your diagnosis with ____ changed your life?

Tell me more about that?

Have you talked to your doctor about how this has affected your life?

What has the doctor said/suggested?

How has this diagnosis changed the way you think about your stomach problems?

How have your symptoms changed since you were diagnosed with ______?

What do you think could happen as a result of having ________?

What does that mean to you?

What are your main concerns about _____ right now?

What are your long-term concerns about ____? OR what do you think you might be concerned about in the future?

***Dysplasia***

Has your doctor ever told you that they found abnormal cells, or dysplasia, during an endoscopy?

If YES

What does this mean to you?

What did the doctor say?

How did this make you feel?

If NO

What would it mean to you if they did find abnormal cells?

How would this make you feel?

Have you ever had a biopsy, cut a piece of the tissue, during an endoscopy?

If YES

Tell me about that?

How did you feel emotionally after the biopsy?

How did you get the results of your biopsy?

What were the results of the biopsy?

How did getting these results make you feel?

If NO

How do you think you would feel emotionally if they had to take a biopsy?

How do you think you would feel getting the results of the biopsy?

***Esophageal Cancer***

Has a doctor ever talked to you about esophageal cancer?

IF YES

Which doctor talked to you about it (primary, specialist)

What did they say about it?

IF NO

What do you know about esophageal cancer?

Where did you get information about esophageal cancer?

Do you think it is a common cancer?

What do you think your chances of developing esophageal cancer are?

When I asked you what your chances are how did you come up with your answer? OR What information do you use when thinking of your chances of developing esophageal cancer?

What would make you change your mind about your chances of developing cancer? What would make you think your chances were higher or lower?

When you think of esophageal cancer what is the first thing you think of?

Have you ever looked for information about esophageal cancer on your own?

What were you looking for?

Where did you look?

How did you feel when you found information?

***Surveillance Endoscopies***

I understand that your doctor has recommended that you have a routine endoscopy.

Can you describe what an endoscopy is and what it’s supposed to do?

Why does your doctor want you to have routine endoscopies?

How did the recommendation for routine endoscopies make you feel? (nervous/relieved?)

Will you tell me more about that? Why did you feel ______?

Did the doctor tell you about things the endoscopy might find?

Probe: understanding of condition/screening for BE/link between BE-EA

What do you think you are at risk of? OR What do you think the doctor thinks you are at risk of?

Did you discuss alternatives with you doctors? Other treatments? Talk about other screening methods—“watchful waiting” or “active surveillance.”

After your first appointment (when you were diagnosed) did you discuss what the doctor told you with anyone?

Who did you talk to? (significant other, family, friends)

What did you talk with them about?

i.e. Talk about the procedure or their concerns?

What did they say or recommend?

Did they want you to have the routine endoscopy?

Were they worried about the possible outcomes

Did they discuss their past experiences with you?

After you spoke to ______ did you feel differently about having the routine endoscopy?

How so? Tell me about that.

Are you planning on having the next routine endoscopy?

Tell me about that.

**If yes**: Why are you choosing to have the next endoscopy?

Probe: desire to get a diagnosis, to rule out BE, improve symptoms?

Are there any other personal reasons you intend to have it?

If: “my doctor told me to”

Probe: Tell me about that. Trust your doctor?

Do you completely trust your doctor’s decisions about which medical treatments are best? Why or why not?

**If no**: Why are you choosing not to have the endoscopy

Do you have concerns specifically about the procedure?

Or possible outcomes of the procedure?

If they say it wasn’t that important to them (low benefit)

Probe: why is it not important?

Do you think you will continue having the routine endoscopies if your doctor recommends them?

What are some reasons that you would stop going to the routine endoscopies?

Do you think that other people with the same diagnosis are having routine endoscopies?

Why do you think they are? Or are not?

Do you think they are having the same amount (number/as often) as you are?

How does this affect your decision?

In what way?

If your spouse’s/loved one’s physician recommended that they have routine endoscopies would you want them to do it?

Why?

If this is a different answer than they gave for themselves then we would probe why they would feel it important for significant other and not themselves.

So you say you plan to have the next endoscopy. Will you describe what you expect to happen on that day?

*Tease out feelings here. They will talk about different steps of this process. For each step:*

Will you say more about how you feel about _______?

ALT: So you say you’re not going to have the next endoscopy. Can you tell me what you would expect to happen if you *did* have the endoscopy? What would happen on that day?

What, do you think, are the benefits to undergoing regular/routine endoscopies?

Continue to probe with “anything else, or can you think of any other benefits”

Did the doctor tell you about benefits of the endoscopy?

Do you think there are any drawbacks to getting the endoscopies?

Continue to probe with “anything else, or can you think of any other drawbacks”

Did the doctor talk about risks of the endoscopies?

How did that information make you feel?

For you is your benefits list or your drawbacks list stronger?

Why?

Did these things help you make your decision to undergo the endoscopy?

In what way?

How did you feel after your last endoscopy? (emotionally and physically)

Did you expect to feel that way?

In terms of your emotions, how do you think you’ll feel after this endoscopy is over?

Will you tell me more about that?

Why do you think you’ll feel that way?

Look at differences

What do you expect to result from the endoscopy?

What will the procedure tell the doctor?

Probe to elicit information about health outcomes (i.e. I could find out if I have abnormal cells (dysplasia), I could improve symptoms.)

*For any outcome they list, probe to elicit feelings* *and what that would mean to them.*

Probe: How does that make you feel?

How do you think the endoscopy might change your understanding of what is going on with your body?

Please tell me more about that?

How does that make you feel?

What do you think could happen if you do NOT have the routine endoscopies?

Probe: In terms of your health, what could happen?

How does that make you feel?

Have you ever had a bad experience during an endoscopy? Tell me about that?

***Barriers***

Have you ever had to miss an endoscopy in the past?

Can you tell me about some of the things that have made it hard for you to keep your endoscopy appointments? (Transportation, prep-fasting, no reminders, no preferences for scheduling, etc)

How had you managed those things? (Rescheduled, called for a ride, took a shuttle/bus, early appt for fasting, etc).

Can you tell me about some of the things that have made it hard to keep appointments that you can NOT manage? (Scheduling, no bus route in area, etc).

Now that we’ve talked about your stomach problems and your routine endoscopies do you feel any differently about going to your next appointment? Or staying on your routine schedule?

**Ask the following questions to patients scheduled for an ablation OR who have recently completed an ablation.**

**IF NO ABLATION SKIP TO MENTAL MODEL**

**Ablation**

***I understand that your doctor: ____ has recommended that you have an ablation***

***____ recently gave you an ablation***

What is an ablation?

Can you walk me through what you expect to happen during your ablation?

**OR if patient is POST-ABLATION*** Can you walk me through your ablation experience?

Why does your doctor want you to have the ablation?

OR Why did your doctor want you to have the ablation?

***If doesn’t mention dysplasia then probe for dysplasia knowledge/understanding***

Did the doctor explain ablation to you?

What did they say about it?

How many ablations have you had?

Have you ever missed an appointment for an ablation?

Why?

What are some of the things you have had to do to keep your ablation appointment?

When the doctor told you that you should have an ablation what was your initial reaction? (How did it make you feel?)

**Do they discuss feelings about possible diagnostic outcomes or do they discuss feelings about the actual procedure? ***

After your appointment (when the doctor told you that you needed an ablation) did you discuss what the doctor told you about the ablation with anyone?

Who did you talk to?

What did you talk to them about?

What did they say or recommend?

Did they want you to have the ablation?

Were they worried about the possible outcomes?

Did they discuss their past experiences with you?

After you spoke to _____ did you feel differently about having the ablation?

How so?

Do you think that other people with the same problem are undergoing ablation?

Why do you think they are? Or are not?

How does this affect your decision?

In what way?

If your spouse’s physician recommended that they have an ablation would you want them to do it?

FOR THOSE PATIENTS SCHEDULED FOR AN ABLATION (if they’ve completed an ablation skip to “completed ablation”

Are you planning on having the ablation? (Yes or no)

If yes: Why are you choosing to have the ablation?

If no: Why are you choosing not to have the ablation?

What, do you think, are the benefits to undergoing the ablation?

Probe with “anything else or can you think of any other benefits”

Did the doctor tell you about benefits of ablation?

What are the drawbacks of undergoing the ablation?

Probe with “anything else or can you think of any other benefits”

Did the doctor tell you about risks of ablation?

In terms of your emotions, how do you think you’ll feel after the ablation is over?

Can you tell me more about that?

Why do you think you will feel that way?

What do you expect to result from the ablation?

What will the ablation tell the doctor?

What do you think the ablation will do for you?

How do you think the ablation might change your understanding of what is going on with your body?

What could happen if you do NOT undergo the ablation?

**IF THEY HAVE COMPLETED AN ABLATION:**

Why did you choose to have the ablation?

What, do you think, are the benefits to undergoing the ablation?

Probe with “anything else or can you think of any other benefits”

Did the doctor tell you about benefits of ablation?

What, do you think, are the drawbacks of undergoing the ablation?

Probe with “anything else or can you think of any other benefits”

Did the doctor tell you about risks of ablation?

In terms of your emotions, how did you feel after the ablation is over?

Can you tell me more about that?

Did you expect to feel that way after the ablation?

What did you expect to result from the ablation?

What will the ablation tell the doctor?

What do you think the ablation will do for you?

Did the ablation change your understanding of what is going on with your body?

What could happen if you did NOT undergo the ablation?

Have you ever had a bad experience after an ablation?

**____________________________________________________________________________**

ASK THE FOLLOWING SECTION TO EACH PARTICIPANT

**Mental Model**

Is this experience with your symptoms, routine endoscopies, (and ablations) similar to any other health problem you have experienced before?

Can you compare this experience to anything (like a health issue or any other event)?

Can you tell me more about that

So now that you have thought through all of these details about your diagnosis, stomach problems and the endoscopies (and ablations). Can you tell me generally what you make of your stomach problems? And how do you feel about the routine endoscopies?

How do you think the way you feel about your stomach problems will change at any point during your endoscopy schedule?

Is there anything else related to your stomach problems or endoscopies that we haven’t covered? Anything you think is important?

THANK YOU _____________ FOR YOUR TIME AND FOR SHARING YOUR EXPERIENCES WITH ME. PLEASE FEEL FREE TO CALL ME IF YOU HAVE ANY QUESTIONS ABOUT THE STUDY. YOU SHOULD RECEIVE YOUR GIFT CARD IN THE MAIL IN A FEW WEEKS.

**Physician Interview**

- **Date of birth** Month___ ___ Year__ __ __ __
- **Gender**   Male  Female
- **Race (Check all that apply)**

White/Caucasian

Black/African American/African-Caribbean/African

American Indian/Alaskan Native

Native Hawaiian or other Pacific Islander

Asian

Hispanic/Latino

Other (specify):____________________________________

- **In what setting do you practice?**

Private

Academic

Veterans Affairs Hospital

- **How long have you practiced in this setting?**

Less than 1 year

1-5 years

5-10 years

10-20 years

20-30 years

>30 years

- **How many years since you completed fellowship?**

Less than 1 year

1-5 years

5-10 years

10-20 years

20-30 years

>30 years

- **How many GERD patients do you treat in a week?** ___________
- **How many Barrett’s Esophagus patients do you treat in a month?** ___________
- **How many esophageal adenocarcinoma patients have you treated in the past year?** ___________
- **How many upper endoscopies do you perform in a week?** ___________

**How many ablations/BARRX do you perform in a month?** ___________

**I: In this interview, we’re going to be talking about your views on scoping different types of patients. We’ll talk about screening for GERD patients, surveillance EGD for BE patients, and ablating patients with different degrees of severity of BE.**

**GERD Patients:**

**For this portion of the interview, I’d like to talk to you about the patients you treat with GERD. Please think about the questions/answers in the context of your patients with GERD and your experiences with screening patients for Barrett’s Esophagus.**

When do you recommend GERD patients have an endoscopy?

-If their decisions are symptom-driven: How long do you typically wait to scope?

Do you think the GERD/BE link is significant?

Tell me about how you see the risk of a GERD patient developing BE.

Can you describe a profile of a patient who needs screening for BE?

-What types?

-What patient characteristics?

-Anything else?

-Does duration of symptoms matter?

What percentage of your patients do you scope specifically for BE?

Now I’d like to do a little role play. Let’s say I’m your patient, I have chronic reflux. And you’ve decided I should have my first endoscopy. Let’s go through what you would tell me in that consultation.

-tease out:

*EGD procedure

*Risk of BE

*Risk of EA

*outcomes of EGD

-if they present something unexpected: “Interesting. What prompted you to say that?”

What is the **take away message** you wanted this patient to leave with?

Please think about a GERD patient that you decided to scope. How did this **patient react** when you suggested they have an EGD? (nervous, relieved, etc)

What questions did he ask you? What are some other questions patients ask?

What do you believe patients think about EGD?

-**Why** do they think they are having EGD?

-Do you feel patients think the EGD is for diagnosis, treatment of symptoms, or some combination?

-Do you feel that patients have an accurate understanding that EGD is a cancer screening tool? (Please explain)

About what percentage of your GERD patients **do NOT follow your recommendation** for an initial screening EGD?

-Why do you think they do not follow the recommendation?

-Can you think of ways to **improve adherence** for GERD patients?

If a GERD patient is scoped and does NOT have BE, what is the treatment plan?

Probe: will you scope them again later?

-If yes: When and how often?

-If no: Why not? Is there a chance they will develop BE later?

-Is this typically what other physicians **in your practice** do?

-Do you think physicians **in other practice settings** use similar guidelines?

**Barrett’s Esophagus Patients:**

**For this portion of the interview, I’d like to talk to you about treating patients with Barrett’s Esophagus. Please think about the questions/answers in the context of patients with Barrett’s Esophagus and your experiences with surveillance endoscopy or repeating endoscopies in patients with Barrett’s Esophagus.**

How has diagnosing BE changed in the last 10 years?

Have you noticed an increase in BE?

-If yes: What do you think is causing this increase in BE diagnoses?

-Is this global or do you think there is something in the U.S.?

-Does it have anything to do with screening practices?

When treating patient with BE, when do you recommend patients undergo surveillance, or repeated endoscopies?

In reviewing patients’ charts for our study, we have noticed that some BE patients do **not have a history of GERD**. What do you think is causing their BE?

How are these patients caught?

(Do they present with other symptoms? At times do they offer EGD with routine colonoscopy even if no symptoms?)

What **types of BE patients** do you tend to recommend enter surveillance plans?

-How often do you recommend they are scoped?

When deciding to recommend a **surveillance plan** for a patient with BE, what is your **frame of reference**? Since there aren’t current practice guidelines.

What do you think is the **standard of care** for surveillance endoscopy in patients with BE?

How do your personal guidelines compare to **your peers’ guidelines/behaviors** at your practice setting?

-How might it **differ in other practice settings**?

Please think about a BE patient you decided to place in a surveillance plan. How did you **explain your recommendation** to have repeated endoscopies?

-Can you walk me through what you said to the patient in the consultation?

-What is the take away message you hope he left with?

How did this **patient react** when you recommend he follow a surveillance plan? Is this a typical reaction?

When patients are in surveillance, does that ever end? For instance, is the BE ever “cured?” How many normal EGD’s should they have to stop surveillance? Do normal EGD’s affect patients’ adherence?

What do you think are the main **reasons that patients decide** to have the recommended surveillance EGD?

What percentage of your BE patients do you believe **do NOT follow your recommendation** for routine EGDs?

-Why do you think they do not follow the recommendation?

-Personal barriers -Systematic barriers

How does adherence change **over time**? For instance, is it common for patients to have several EGDs and then stop coming?

-If yes: Why does this happen?

In your opinion, what could be done to **improve the adherence rates** for BE patients?

Is there a **difference in adherence rates** for patients with GERD and patients with BE?

-If yes: What is the difference? Why do you think there is a difference?

Can you tell me about any system-level barriers you have experienced that have affected your ability to do EGDs?

**Ablation:**

I understand that ablations/BARRX or RFA are performed here.

Do you conduct these procedures?

Can you tell me about that procedure?

Will ablation get rid of the dysplasia in the ablated area permanently, or can it recur?

When do you recommend patients undergo ablation or BARRX (RFA)?

-Do you recommend ablation for low- and high-grade dysplasia? Why?

Please think about a recent time when you decided to perform/recommend ablation/RFA on a patient. How did you explain the procedure? What did you say about dysplasia?

How did this patient react when you recommend he have ablation? Is this typical?

What do you think is the patients’ perspective as to why they are having the ablation?

-Do they understand the benefits/risks of ablation?

Does non-adherence to ablation tend to be a problem? Why or why not?

**Now I’d like to talk with you a bit about esophageal cancer:**

How do you see the **risk** of a person with BE developing esophageal cancer?

What are some **risk factors** for developing esophageal adenocarcinoma?

Considering the (low) risk of a patient developing esophageal adenocarcinoma, do you think it’s **worthwhile to scope** <their reported standard of care>?

In thinking about the link between Barrett’s Esophagus and esophageal adenocarcinoma, what other precancerous condition would you compare BE to?

When do you talk to patients about their cancer risk?

-Do you talk about cancer risk with GERD patients? BE patients? At what point?

-What do you say about cancer risk?

Will you tell me about an experience you’ve had (good or bad) that has changed the way you provide care in GERD or BE patients?

As technology advances, how do you see practice guidelines changing (frequency, more ablations, etc)? How do you see future technological advances affecting patients’ adherence?

Framework Analysis: Coding Key for Patient Interviews

Cause of GERD/BE

Physical

-exertion

Chemical

-acid

-preservatives

-other medications (diabetes med)

-exposures (Agent Orange)

Psychological

-stress

-nervousness

-thought processes

Confusion about causality/ negotiation of causality

Triggers of GERD

Behavioral triggers

-food (type of food)

-overeating

-forgot to take meds

Psychological triggers

-stress

-exertion

Knowledge/understanding of BE

Prevalence

-uncommon

-more common than we realize

Terminology

-stages/steps/levels

Outcome expectancies

-curability vs. management

-trajectory

-severity (BE=cancer)

Misperceptions

-acid caused a hole in esophagus

-acid ate away the flap that closes the stomach

Quality of Life

Health-related QoL

-symptoms are scary (can’t swallow)

-suicidal because symptoms severity

-diet changes b/c of symptoms

-aggravation of using medications

General QoL

-relationship with spouse, family, friends

-presentation of self (teeth, scars)

-embarrassing

Symptom management

Pharmaceutical

-OTC

-Rx

-descriptions of effectiveness/problems

Behavioral/self-management

-pillows, diet, vomiting, weight loss, humidifier, water, breathing

-descriptions of effectiveness/problems

Symptoms

Description of symptoms/illness presentation

-pain, burning

Duration

-onset and changes to symptoms over the years

Severity

-little bother to extreme bother

Outcomes of symptoms

-dental problems

-hernia

No symptoms

Reasons for initial EGD

BE study

Symptoms

Reaction to EGD recommendation

Emotional responses

-relief

-reassurance

-hopeful

-recommended frequency is a way to self-monitor their health

-curious

-intrusive/invasive

-suspicious about efficacy

-fear (could be fatal)

-anxiety

Heuristics

-colonoscopy

Outcome expectancy of EGD

Physical

-sore

-drowsy

-damage to esophagus

-feel fine/feel nothing

Cognitive

-curious of findings

-immediate assessment of health

-empowerment (to monitor condition, control)

-nothing will be different

-it will show improvement

Experience of EGD

-not a big deal

-pain or problems

-not enough anesthesia

-infection from biopsy

Reasons for follow-up

Monitoring

-watch for cancer

-see if med is working

Curative

-get rid of BE

Emotional

-defer responsibility

-sense of control

Reaction to diagnosis

-uncommon

-strange/unfamiliar

-doesn’t make sense, never had GERD symptoms

-anger

-blame self/responsibility

-scared

-kept it secret to not worry loved ones

-became more spiritual

-distance self

-not worried, not a big deal

-glad (empowerment, management)

-same as GERD

-confirmed suspicions that something was wrong

Ablation

Experiences

-Pain/discomfort

-no pain

Emotions

-felt good to have it over with, control disease

-doesn’t have to worry about cancer now

Outcome expectancy/ Reason for adherence

-cure BE

-prevent cancer

Esophageal cancer

Reaction

-uncommon

-fatal

Perceived risk

-factors that increase risk

-behavioral- diet

-genetics

-factors that decrease risk

-genetics

Confusion

-EA=GERD=BE

Heuristics

-other family or friends with cancer

Barriers

Emotional

-bad experience/fear of procedure

-lack of trust in doctors

Structural

-transportation

-gas prices

-other appointments

-work

-parole meetings

-VA system

-burden of driving to VA

Facilitators

-scheduling options

-shuttle, info on transportation options

-social support (spouse, church)

-VA coverage/no cost treatment

Opinion of docs

-trust

-reliance

-specialist vs. pcp

-opinion of MEDVAMC

-doc is personally invested

-not thorough
